# Performance of a methylation specific real-time PCR assay as a triage test for HPV-positive women

**DOI:** 10.1186/s13148-017-0419-2

**Published:** 2017-10-24

**Authors:** Martina Schmitz, Kristina Wunsch, Heike Hoyer, Cornelia Scheungraber, Ingo B. Runnebaum, Alfred Hansel, Matthias Dürst

**Affiliations:** 1 oncgnostics GmbH, Jena, Germany; 2 Department of Gynaecology and Reproductive Medicine, Jena University Hospital, Jena, Germany; 3 Institute of Medical Statistics, Information Sciences and Documentation, Jena University Hospital, Jena, Germany; 4 Department of Gynaecology and Reproductive Medicine, Jena University Hospital, Am Klinikum 1, 07747 Jena, Germany

**Keywords:** Screening, CIN, Cervical cancer, HPV, Triage, Methylation marker panel

## Abstract

**Background:**

HPV DNA testing as a primary screening marker is being implemented in several countries. Due to the high HPV prevalence in the screening population, effective triage strategies for HPV-positive cases are required. The aim of this study was to evaluate the performance of a methylation-specific real-time PCR  assay (GynTect®) comprising six marker regions as a triage test.

**Results:**

An analytical sensitivity of 0.1 ng genomic DNA corresponding to 15 SiHa cells was achieved. Absolute specificity was observed in the presence of 20 ng unmethylated genomic DNA. In a clinical setting, cervical scrapes of 306 women showing abnormal colposcopy were tested for cytology, HPV positivity, and the GynTect markers ASTN1, DLX1, ITGA4, RXFP3, SOX17, and ZNF671. Of all women, histopathological data were available. The overall sensitivity for GynTect to detect CIN3+ was 67.7% (95% CI 57.3%–77.1%) whereas sensitivity was significantly higher for women of age ≥ 30 years (*p* = 0.04). All cancer cases (*n* = 5) were detected by GynTect. The overall false positive rate (= 1-specificity) for women with no CIN was 17.4% (95% CI 12.5–23.1%), with a higher proportion among HPV-positive women (24.0%, 95% CI 16.0–33.6%). In a triage screening setting, where all women underwent HPV testing and the HPV positives in addition GynTect testing, the overall sensitivity would slightly decline but specificity would reach the maximum value of 88.7% (95% CI 83.7–92.6%).

**Conclusion:**

The GynTect® assay is a robust easy to use assay with high analytical sensitivity and specificity. Moreover, the performance of the assay based on cervical scrapes provides further evidence for the usefulness of methylation markers to detect HPV-positive women with clinically relevant disease.

**Electronic supplementary material:**

The online version of this article (10.1186/s13148-017-0419-2) contains supplementary material, which is available to authorized users.

## Background

Persistent infection with high-risk human papillomaviruses (HPV) is a prerequisite for the development of cervical precancerous lesions and cancer [1]. Not surprisingly, testing for HPV-DNA in a cervical cancer screening setting is highly sensitive for the detection of clinically relevant lesions. Indeed, a follow-up study of four randomized trials in which HPV-based screening was compared with cytology-based screening (with precancerous lesions as endpoint) showed that HPV-based screening provided 60–70% greater protection for incident invasive cervical carcinomas compared with cytology [2].

The disadvantage of HPV-based screening is the high number of women who are HPV positive without having a disease. This is especially the case for women age 30 years and younger [3]. Triage strategies for HPV-positive cases are therefore required to identify women in need of treatment. Beside Pap staining and immunostaining for p16/Ki67, methylation markers have shown great promise as triage tools in cervical cancer diagnostics [4]. Clearly, implementation of an efficient triage strategy will reduce unnecessary colposcopies and invasive diagnostic work-up.

In previous work, we have identified a marker panel comprising six different regions rich in cytosines and guanines and, especially, in cytosine-guanine dinucleotides, so-called CpG islands, in the human genome which were frequently methylated in cervical precancers and cancer cases [5]. Based on these methylation markers and two internal controls, we have recently developed GynTect®, a CE-IVD-certified assay for the sensitive and specific detection of these marker regions. In the present report, we investigated the analytical and clinical performance of this new assay using cervical scrapes suspended in STM™ buffer which is the transport medium for the QIAGEN Hybrid Capture II HPV test.

## Methods

### Cell culture

The HPV16-positive cervical cancer cell line SiHa (ATCC^®^ HTB-35^™^) was used as positive control and for determining the analytical sensitivity and PCR efficiency. SiHa cells were cultured in Dulbecco’s modified Eagle medium supplemented with 10% fetal calf serum, 100 units/ml penicillin, and 100 μg/ml streptomycin in a humidified incubator at 37 °C with 5% CO_2_.

### Ethics statement

This work was approved by the ethics committee of the Friedrich Schiller University Jena (Reference numbers 2174-12/07 and 3471-06/12). All patients provided written informed consent to use their cervical cell scrapes and the corresponding clinicopathological data for molecular analyses.

### Cervical scrapes

Study population: Cervical cell scrapes were obtained from women visiting the colposcopy unit in the Department of Gynaecology at Jena University Hospital between November 2013 and June 2015. Cell scrapes were collected in 4 ml PBS (pH = 7.4). Two milliliters of the cell suspension were centrifuged at 1000*g*, and the pellet was resuspended in 0.5 ml Specimen Transport Medium (STM™, Qiagen, Hilden, Germany) for short-term storage at + 4 °C and subsequent GynTect analysis.

For all patient, the HPV status was determined using the GP5+/6+ PCR-EIA assay [6]. For this purpose, the sample in PBS was used.

Routine Pap smears were collected for most patients, and cytologic findings were reported according to the Munich II/III nomenclature. Pap III (Bethesda System: ASC-H) or worse was defined as being positive.

### DNA isolation and bisulfite treatment

DNA was isolated from cultured SiHa cells using the NucleoSpin® Tissue Kit (Macherey Nagel, Düren, Germany) following the instructions of the manufacturer.

For bisulfite treatment of purified genomic DNA, the DNA Methylation Gold Kit (Zymo Research Europe, Freiburg, Germany) was used according to the manufacturer’s protocol. Concentration of bisulfite-treated DNA was measured using a Nanodrop 2000 UV-Vis spectrophotometer (PeqLab (VWR Life Science), Erlangen, Germany). For bisulfite treatment of cervical samples, the EpiTect Fast Bisulfite Kit (Qiagen) was used. Forty microliters of the cervical sample in STM™ sample buffer was directly used for bisulfite treatment without prior DNA isolation. This volume corresponds to 4% of the entire cervical sample. After elution in 20 μl elution buffer, 70 μl water was added, and 10 μl of the diluted DNA were used for each single reaction in the GynTect real-time PCR assay.

Analytical sensitivity of the assay was determined with and without unmethylated DNA as background. To obtain fully methylated DNA, DNA from SiHa cells was in vitro methylated using CpG-Methyltransferase (M.SssI) from New England Biolabs according to protocol. Then, fully methylated SiHa DNA was bisulfite treated and diluted in water down to 1 pg as total amount of input DNA (6–7 pg correspond to the DNA content of approx. one cell). Moreover, assay sensitivity was also determined for methylated DNA in a background of unmethylated DNA. For this purpose, a dilution series of bisulfite-treated SiHa DNA (10, 5, 2, 1, 0.5, 0.2%) in a background of bisulfite-treated control DNA extracted from HPV negative, cytologically normal cervical scrapes was used. A total amount of 20 ng bisulfite-treated DNA was analyzed in each PCR reaction. Amplification results for the internal control marker ACHE were a measure for equal amount of input DNA in all PCR runs. Dilution series were set up in three different experiments, and each experiment was performed in triplicate.

To determine the analytical specificity, EpiTect Control DNA (Qiagen, Hilden, Germany) was bisulfite treated with the DNA Methylation Gold Kit (Zymo Research Europe, Freiburg, Germany). DNA was eluted in 20 μl elution buffer, and concentration was measured using a Nanodrop 2000 UV-Vis spectrophotometer (PeqLab (VWR Life Science), Erlangen, Germany). Twenty and 100 ng of bisulfite-treated EpiTect Control DNA was analyzed in three different experiments, and each experiment was performed in triplicate.

### Methylation-specific PCR (GynTect®)

The GynTect® assay (oncgnostics GmbH, Jena, Germany) is based on the methylation panel for the detection of CIN2+ published by Hansel and colleagues [5]. The assay was CE-certified for the use of STM™ (QIAGEN, Hilden, Germany) in 10/2015 and requires no specialized bioinformatic algorithm. PCR is performed using oncgnostics custom-made real-time PCR mastermix containing a hotstart DNA polymerase. As quality control, bisulfite-specific primers targeting a region close to the ACHE gene locus devoid of CpG dinucleotides were used and, as a methylation control, methylation-specific primers targeting a methylated region of the imprinted IDS gene located on the X-chromosome. For all primer pairs, different concentrations were used. Each marker is detected in a singleplex real-time PCR using EvaGreen (Biotium, USA) as intercalating dye. For all samples, PCR of the single marker regions is performed in the individual tubes of an eight-well strip in which the primers are pre-dried for the respective markers. For running the real-time PCR, 10 μl mastermix and 10 μl sample was added to each tube.

Real-time PCRs were performed using the ABI7500 PCR system (Life Technologies, Thermo Fischer Scientific, USA). After a 1-min period at 94 °C, 42 cycles at 94 °C for 15 s and 67 °C for 30 s were run, followed by a standard melting curve. PCR runs were analyzed using the ABI Software V2.0.6 and Excel 2007. Samples were considered to be of sufficient quality if the Ct value for the quality control marker ACHE was ≤ 35. Samples were scored methylation positive for each marker region, if a PCR product characterized by its typical melting curve determined directly after methylation-specific PCR amplification was obtained within 42 cycles. The whole GynTect assay was considered to be positive if the added score of all six marker regions was 0.5 or higher. The scores for the single markers are 0.5 for ZNF671; 0.2 for each of the markers ASTN1, ITGA4, RXFP3, and SOX17; and 0.1 for DLX1. ZNF671 has the best specificity among the six markers, but sensitivity for ZNF671 alone is not sufficient in a clinical context; therefore, the other five markers are used to improve sensitivity.

### Statistical analysis

The test positive rates were calculated for cytology (Pap), HPV, and GynTect according to age group (< 30 years vs ≥ 30 years) and histology of the corresponding biopsy sample. Sensitivity and specificity were estimated to evaluate the diagnostic performance. Exact 95% confidence intervals (CI) were calculated for the proportions assuming a binomial distribution. In addition to the single test performance, a triage test result was evaluated considering women with positive results in both HPV and GynTect testing as triage positive, everything else as triage negative. Age groups were statistically compared by the *χ* ^2^ test, the level of significance was set to 0.05.

## Results

### Analytical sensitivity for dilution series of methylated DNA in water

Reproducible amplification of all methylation-specific marker regions and controls was achieved with 0.1 ng bisulfite-treated DNA (corresponding to approx. 15 tumor cells). Results are summarized in Table 1.

**Table 1:** Analytical sensitivity, determined in three independent experiments, each in triplicate

Input DNA	Corresponding cells^a^	ASTN1	DLX1	ITGA4	RXFP3	SOX17	ZNF671	ACHE	IDS
0.3 ng	45 cells	n.d.	n.d.	n.d.	n.d.	n.d.	n.d.	n.d.	9/9
0.2 ng	30 cells	n.d.	n.d.	n.d.	n.d.	9/9	n.d.	n.d.	9/9
0.1 ng	15 cells	9/9	9/9	9/9	9/9	9/9	9/9	9/9	9/9
0.05 ng	7.5 cells	9/9	9/9	9/9	9/9	6/9	9/9	9/9	6/9
0.025 ng	4 cells	9/9	8/9	8/9	8/9	5/9	8/9	4/9	4/9
0.01 ng	1.5 cells	5/9	6/9	5/9	5/9	2/9	5/9	2/9	1/9
0.005 ng	0.7 cells	3/9	4/9	0/9	3/9	1/9	4/9	2/9	1/9
0.002 ng	0.3 cells	0/9	0/9	1/9	2/9	0/9	1/9	0/9	n.d.
0.001 ng	0.15 cells	1/9	2/9	2/9	1/9	n.d.	0/9	0/9	n.d.

*n.d.* not done

^a^Assuming one cell contains 6-7 pg DNA

The analytical sensitivity for the detection of methylated DNA in a background of unmethylated DNA instead of water was 0.5% methylated DNA (corresponding to ca. 15 cells) for all six marker regions (Table 2). Except SOX17, all marker regions and the quality control ACHE could reproducibly be amplified in a 0.2% dilution (corresponding to ca. 6 cells). IDS showed the lowest sensitivity having a detection limit of 2% methylated DNA. Results are shown in Table 2.

**Table 2:** Analytical sensitivity in a background of unmethylated DNA, determined in three independent experiments, each in triplicate

Proportion of methylated DNA in 20 ng input DNA	Amount of methylated DNA	ASTN1	DLX1	ITGA4	RXFP3	SOX17	ZNF671	ACHE	IDS
10%	2 ng	9/9	9/9	9/9	9/9	9/9	9/9	9/9	9/9
5%	1 ng	9/9	9/9	9/9	9/9	9/9	9/9	9/9	9/9
2%	0.4 ng	9/9	9/9	9/9	9/9	9/9	9/9	9/9	9/9
1%	0.2 ng	9/9	9/9	9/9	9/9	9/9	9/9	9/9	8/9
0.5%	0.1 ng	9/9	9/9	9/9	9/9	9/9	9/9	9/9	8/9
0.2%	0.04 ng	9/9	9/9	9/9	9/9	8/9	9/9	9/9	7/9

For all six markers and IDS, unmethylated, bisulfite-converted DNA was taken as background DNA for all primer pairs

**Fig. 1:**
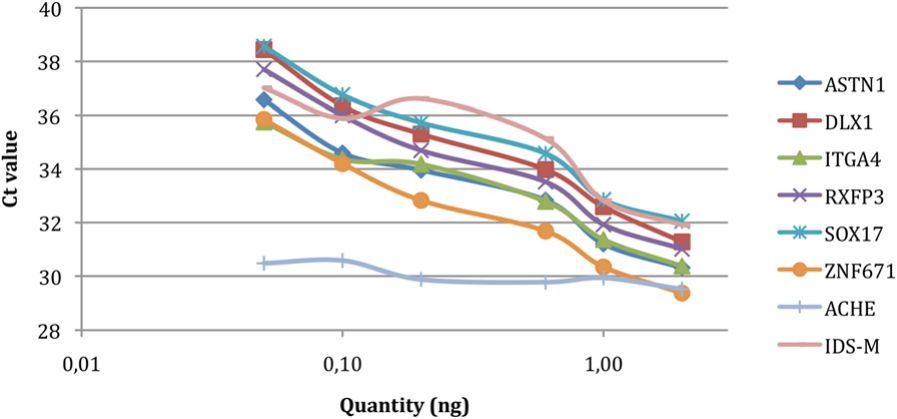
Standard curves of all markers and IDS tested on a dilution series of SiHa DNA in unmethylated DNA as background. Determination coefficient R2: ASTN1: 0.97005, DLX1: 0.982, ITGA4: 0.9679, RXFP3: 0.98465, SOX17: 0.97894, ZNF671: 0.98631, and IDS: 0.84382

The standard curves for each primer pair using the mean Ct values from all nine PCR runs shown in Table 2 are summarized in Fig. 1. All primer pairs showed a very good determination coefficient with > 0.96 for the methylation marker and > 0.84 for the methylation control IDS. The ACHE curve shows that equal amounts of total DNA were used to determine the analytical sensitivity in a background of unmethylated DNA.

### Analytical specificity

For analytical specificity, 20 and 100 ng unmethylated, bisulfite-treated DNA was used as input amount for PCR. Regarding 100 ng input DNA, one positive result was obtained for RXFP3 and ZNF671 (Table 3). To determine the analytical specificity for ACHE, genomic DNA (20 and 100 ng, each in ninefold determination) was used as input DNA. For two markers, a positive result was obtained in one reaction (Table 3).

**Table 3:** Determination of the specificity of GynTect

Amount of DNA	ASTN1	DLX1	ITGA4	RXFP3	SOX17	ZNF671	ACHE	IDS
20 ng unmethylated DNA	0/9	0/9	0/9	0/9	0/9	0/9	9/9	0/9
100 ng unmethylated DNA	0/9	0/9	0/9	1/9	0/9	1/9	9/9	0/9

Two false positives are detected for RXFP3 and ZNF671

### Clinical performance

In order to demonstrate the diagnostic potential of the GynTect markers for the detection of CIN3+ among women of all age groups, a cross-sectional study was performed for patients referred to the colposcopy unit of the Department of Gynaecology at Jena University Hospital. Only women with a colposcopic indication for biopsy and thus a histopathological diagnosis were included. Of the 306 women included in the cohort, 199 were 30 years and older. For all patients, the HPV status was determined using the GP5+/6+-EIA assay [6]. The Pap test was not done, or the result was not valid for 22 patients. Thus, 131 out of 284 (46.1%, 95% CI 40.2–52.1%) were tested Pap III or worse (Table 4). Out of all 306 samples, 189 (61.8%, 95% CI 56.1–67.2%) were tested HPV positive (Table 5). Out of all 306 samples, 100 (32.7%, 95% CI 27.5–38.2%) were tested GynTect positive (Table 6).

**Table 4:** Cytological test positive rate (Pap III+) according to histology and age

Age	Pap III+	Histology				Total
		noCIN	CIN 1-2	CIN 3	CxCa	
< 30	Rate [%]	21.7	69.6	74.2		49.0
	95% CI [%]	10.9–36.4	47.1–86.8	55.4–88.1	–	38.9–59.2
	Count	(10/46)	(16/23)	(23/31)	–	(49/100)
≥ 30	Rate [%]	20.0	57.7	86.3	100	44.6
	95% CI [%]	12.8–28.9	36.9–76.6	73.7–94.3	22.4–100	37.3–52.1
	Count	(21/105)	(15/26)	(44/51)	(2/2)	(82/184)
Total	Rate [%]	20.5	63.3	81.7	100	46.1
	95% CI [%]	14.4–27.9	48.3–76.6	71.6–89.4	22.4–100	40.2–52.1
	Count	(31/151)	(31/49)	(67/82)	(2/2)	(131/284)

Cytology missing/not done: *n* = 22

**Table 5:** High-risk HPV test positive rate (all 14 hrHPV types) according to histology and age

Age	HPV+	Histology				Total
		noCIN	CIN 1–2	CIN 3	CxCa	
< 30	Rate [%]	49.0	84.0	96.9	100	72.0
	95% CI [%]	34.4–63.7	63.0–95.5	83.8–99.9	5.0–100	62.5–80.2
	Count	(24/49)	(21/25)	(31/32)	(1/1)	(77/107)
≥ 30	Rate [%]	30.1	80.8	94.6	100	56.3
	95% CI [%]	21.8–39.4	60.6–93.4	85.1–98.9	47.3–100	49.1–63.3
	Count	(34/113)	(21/26)	(53/56)	(4/4)	(112/199)
Total	Rate [%]	35.8	82.4	95.5	100	61.8
	95% CI [%]	28.4–43.7	69.1–91.6	88.8–98.7	54.9–100	56.1–67.2
	Count	(58/162)	(42/51)	(84/88)	(5/5)	(189/306)

hrHPV+ refers to the presence of one or more of the following HPV types: HPV16, 18, 31, 33, 35, 39, 45, 51, 52, 56, 58, 59, 66, and 68

**Table 6:** GynTect test positive rate according to age and histology

Age	GynTect+	Histology				Total
		noCIN	CIN 1–2	CIN 3	CxCa	
< 30	Rate [%]	10.2	12.0	53.1	100	24.3
	95% CI [%]	3.4–22.2	2.5–31.2	34.7–70.9	5.0–100	16.5–33.5
	Count	(5/49)	(3/25)	(17/32)	(1/1)	(26/107)
≥ 30	Rate [%]	17.7	34.6	73.2	100	37.2
	95% CI [%]	11.2–26.0	17.2–55.7	59.7–84.2	47.3–100	30.5–44.3
	Count	(20/113)	(9/26)	(41/56)	(4/4)	(74/199)
Total	Rate [%]	15.4	23.5	65.9	100	32.7
	95% CI [%]	10.2–21.9	12.8–37.5	55.0–75.7	54.9–100	27.5–38.2
	Count	(25/162)	(12/51)	(58/88)	(5/5)	(100/306)

**Fig. 2:**
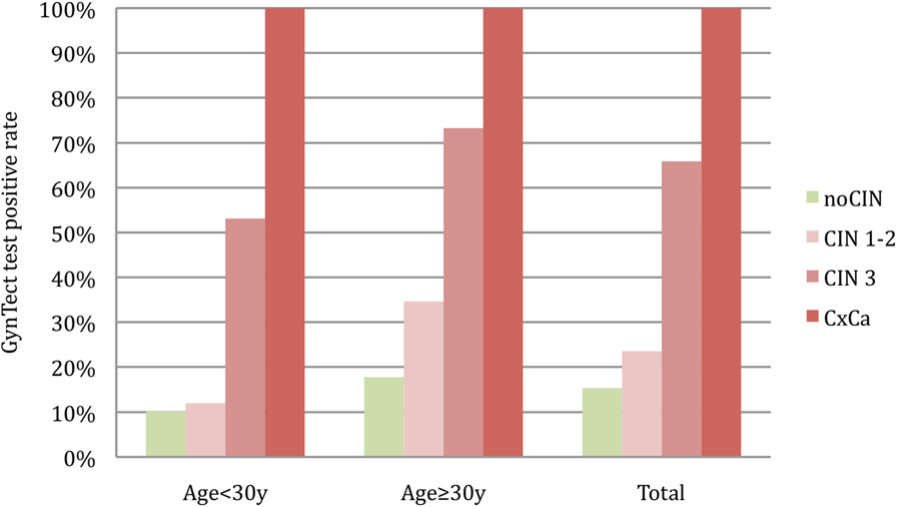
GynTect test positive rate according to histology and age

All five cancer cases were detected by GynTect. Sixty-six percent (58/88) of the CIN3 cases and 23.5% (12/51) of the CIN 1–2 cases were GynTect positive. In the “no CIN” group, 15.4% (25/162) were GynTect positive. The test results according to age and histology are summarized in Table 6 and Fig. 2.

The overall false positive rate (= 1-specificity) for women with no CIN was 17.4% (95% CI 12.5–23.1%), with a higher proportion among HPV-positive women (24.0%, 95% CI 16.0–33.6%).

Table 7 shows the diagnostic performance for detection of CIN3 or cervical cancer (CIN3+) in terms of sensitivity and specificity. Raw data underlying these estimates are presented in Additional file 1: Table S1. The overall sensitivity of the single GynTect is 67.7% (95% CI 57.3–77.1%), the specificity is 82.6% (95% CI 76.9–87.5%). Sensitivity is significantly higher for women in the age group ≥ 30 than in the age group < 30 years (75.0 vs 54.5%, *p* = 0.04), whereas specificity is about 10% lower in the age group ≥ 30 compared to younger women (79.1 vs 89.2%, *p* = 0.07). In the subgroup of HPV test-positive women specificity of GynTect decreased to 76% without remarkable change in sensitivity. In a triage screening setting, where all women underwent HPV testing and the HPV positives in addition GynTect testing, the overall sensitivity would slightly decline but specificity would reach the maximum value of 88.7% (95% CI 83.7–92.6%). For comparison, the Pap test overall performance is 82.1% (95% CI 72.3–89.6%) for sensitivity and 69.0% (95% CI 62.1–75.3%) for specificity (Table 7).

**Table 7:** Diagnostic performance of Pap test (missing/not done: *n* = 22), HPV and GynTect as single tests and triage by HPV and GynTect for the detection of CIN3 or cervical cancer (CIN3+)

		Age < 30		Age ≥ 30		Total	
		Sensitivity CIN3+	Specificity CIN3+	Sensitivity CIN3+	Specificity CIN3+	Sensitivity CIN3+	Specificity CIN3+
Pap III+	Estimate [%]	74.2	62.3	86.8	72.5	82.1	69.0
	95% CI [%]	55.4–88.1	49.8–73.7	74.7–94.5	64.0–80.0	72.3–89.6	62.1–75.3
	Count	(23/31)	(43/69)	(46/53)	(95/131)	(69/84)	(138/200)
HPV	Estimate [%]	97.0	39.2	95.0	60.4	95.7	53.1
	95% CI [%]	84.2–99.9	28.0–51.2	86.1–99.0	51.8–68.6	89.4–98.8	46.1–59.9
	Count	(32/33)	(29/74)	(57/60)	(84/139)	(89/93)	(113/213)
GynTect	Estimate [%]	54.5	89.2	75.0	79.1	67.7	82.6
	95% CI [%]	36.4–71.9	79.8–95.2	62.1–85.3	71.4–85.6	57.3–77.1	76.9–87.5
	Count	(18/33)	(66/74)	(45/60)	(110/139)	(63/93)	(176/213)
GynTect in HPV positives	Estimate [%]	56.3	84.4	73.7	69.1	67.4	76.0
	95% CI [%]	37.7–73.6	70.5–93.5	60.3–84.5	55.2–80.9	56.7–77.0	66.4–84.0
	Count	(18/32)	(38/45)	(42/57)	(38/55)	(60/89)	(76/100)
HPV and GynTect (Triage)	Estimate [%]	54.5	90.5	70.0	87.8	64.5	88.7
	95% CI [%]	36.4–71.9	81.5–96.1	56.8–81.2	81.1–92.7	53.9–74.2	83.7–92.6
	Count	(18/33)	(67/74)	(42/60)	(122/139)	(60/93)	(189/213)

Triage results are defined as negative if HPV negative or HPV positive but GynTect negative and defined as positive in case of HPV positive and GynTect positive

## Discussion

Here, we describe the performance of a new commercially available assay, which was developed as a triage tool for detecting severe cervical lesions (CIN3) and cervical cancer in HPV-positive women. The absolute analytical sensitivity of all markers included in the assay called GynTect corresponds to DNA from approximately seven to eight cells for all markers except SOX17 and IDS, for which the detection limit is 15 cells. The six methylation biomarkers are detected reliably down to a dilution step of 0.5% in a background of unmethylated DNA (corresponding to 15 positive cells). By projecting the analytical performance of GynTect to clinical samples, of which GynTect is using 4% for the analysis, a minimum of 375 methylation-positive cells would have to be present in the entire cervical scrape in order to be scored positive. False negative results due to an insufficient number of dysplastic cells are therefore highly unlikely.

The overall sensitivity for the detection of CIN3 or cervical cancer (CIN3+) was 67.7% (95% CI 57.3–77.1%). Of note is that all cancer cases were detected by GynTect. We used CIN3+ rather than CIN2+ to determine the clinical performance because of the heterogeneous classifications when diagnosing CIN2 [7] and the high regression rate of CIN2 especially among young women [8].

The overall false positive rate (= 1-specificity) for women with no CIN was 17.4% (95% CI 12.5–23.1%), with a higher proportion among HPV-positive women (24.0%, 95% CI 16.0–33.6%). It should be noted that our study population comprised exclusively women who were referred to our colposcopy unit for diagnostic work-up. Although histopathology is the diagnostic gold standard, we cannot exclude biopsy sampling error or occult endocervical disease among these patients probably resulting in rare cases of histological misclassification. Moreover, our clinical sample collection does not represent a primary screening population, and the diagnostic performance measures reported here are not applicable to primary screening. In particular, this applies to the “no CIN” subgroup and the false positive rates.

To date, several other methylation markers were reported to show a high potential for triaging HPV-positive women [9–13]. Compared to other methylation markers, such as CADM1, MAL, miR124, FAM19A4, [11, 14, 15] or PAX-1, ZNF582, SOX1, and NKX6-1 [16], the GynTect assay shows a comparable sensitivity but a higher specificity. Tian and colleagues propose a combined screening algorithm with HPV: HPV 16/18-positive cases go directly to colposcopy whereas hrHPV positives other than 16/18 are triaged by the methylation markers (PAX1 and/or ZNF582). In the described cohort, a sensitivity (CIN3+) of 78.85% and a specificity of 73.55% was achieved [16]. Possibly, by adding more markers to the GynTect® panel, specificity could be improved even further but this would most likely lower sensitivity. Other aspects that would need to be considered when increasing the number of markers to be included in a panel are assay practicability and increased costs.

Compared to other triage markers such as CINtec Plus which is based on p16/Ki67 immunostaining, the GynTect assay has a lower sensitivity for CIN3+ (67.7%, 95% CI 57.3–77.1%) (CINtec Plus: sensitivity between 93.2 and 96.4% [17–19]). GynTect displays, however, a very high specificity of 82.6% (95% CI 76.9–87.5%), which is much higher than for p16/Ki67 dual staining which in previous studies ranged between 46.1 and 76.9% [17–19]. p16/Ki67 is a suitable tool to reliably detect CIN3+, but in a screening setting with a preselection based on a highly sensitive test such as HPV DNA or mRNA detection, p16/ki67 does not significantly reduce the number of women to be referred to colposcopy. A test, which is more specific but, on the other hand, does not miss any critical disease cases would be ideal. Moreover, overtreatment is still a major issue due to side effects of invasive therapy such as perinatal loss, preterm birth, bleeding, long-term absence from work, and mental stress [20–22]. In previous work, spontaneous regression of CIN2/3 lesions to normal histopathology was shown to be as high as 30% within 36 weeks without any treatment [23]. In another study which tested the efficacy of Imiquimod in the treatment of CIN2/3, the regression rate in the placebo group was 39% within 16 weeks (11 of 28 patients) [24]. This is in line with other publications showing that not all CIN3 are progressing to cancer [25]. In this context, it is noteworthy that the proportion of GynTect-positive CIN3 lesions correspond to the proportion of persisting CIN3 lesions in the above studies. It is therefore tempting to hypothesize that GynTect detects all relevant CIN3 lesions in need of treatment. Furthermore, the GynTect detection rate in CIN irrespective of lesion grade shows age dependence. In younger women < 30 years, considerably fewer lesions are GynTect positive. As is shown in Table 6, the GynTect-positive rate among women < 30 years diagnosed with CIN1/2 was 12%, which again is in line with results of a recent study performed by Loopik et al., which showed that the CIN1/2 regression rate among young women < 25 years is > 70%, and the progression rate to CIN3 as low as 15% [8].

## Conclusion

GynTect® is a robust and highly reproducible assay for the triage of HPV-positive women. Further studies are required to determine the clinical outcome of GynTect-negative CIN2/3 lesions, particularly in young women.

## Additional file

Additional file 1: Table S1. Distribution of combined test results according to histology and age. (DOCX 37 kb)

## Abbreviations

ACHE: Acetylcholinesterase
ASTN1: Astrotactin 1
CADM1: Cell adhesion molecule 1
CIN: Cervical epithelial neoplasia
DLX1: Distal-less homeobox 1
FAM19A4: Family with sequence similarity 19 member A4
HPV: Human papilloma virus
hrHPV: High-risk HPV
IDS: Iduronate 2-sulfatase
ITGA4: Integrin subunit alpha 4
MAL: T cell differentiation protein
miR124: MicroRNA 124
NKX6–1: NK6 homeobox
PAX-1: Paired box 1
PCR: Polymerase chain reaction
RXFP3: Relaxin/insulin like family peptide receptor 3
SOX1: SRY-box 1
SOX17: SRY-box 17
ZNF582: Zinc finger protein 582
ZNF671: Zinc finger protein 671
