# Supplementary material for: Performance of a methylation specific real-time PCR assay as a triage test for HPV-positive women
Source: Clin Epigenetics. 2017 Oct 24;9:118. doi: 10.1186/s13148-017-0419-2 (PMC5655856; doi:10.1186/s13148-017-0419-2)
Supplement: Additional file 1: Table S1. — Distribution of combined test results according to histology and age. (DOCX 37 kb) [file 13148_2017_419_MOESM1_ESM.docx]

Additional file 1: Table S1. Distribution of combined test results according to histology and age

|  |  |  |  |  | **Histology** | | | | |
| --- | --- | --- | --- | --- | --- | --- | --- | --- | --- |
| **Age** | **Pap III+** | **HPV** |  |  | **noCIN** | **CIN 1-2** | **CIN 3** | **CxCa** | **Total** |
| <30 y | missing | negative | **GynTect** | negative | 1 |  |  |  | 1 |
|  |  |  | Total | | 1 |  |  |  | 1 |
|  |  | positive | **GynTect** | negative | 2 | 2 | 0 | 0 | 4 |
|  |  |  |  | positive | 0 | 0 | 1 | 1 | 2 |
|  |  |  | Total | | 2 | 2 | 1 | 1 | 6 |
|  |  | Total | **GynTect** | negative | 3 | 2 | 0 | 0 | 5 |
|  |  |  |  | positive | 0 | 0 | 1 | 1 | 2 |
|  |  |  | Total | | 3 | 2 | 1 | 1 | 7 |
|  | negative | negative | **GynTect** | negative | 20 | 2 | 1 |  | 23 |
|  |  |  |  | positive | 1 | 0 | 0 |  | 1 |
|  |  |  | Total | | 21 | 2 | 1 |  | 24 |
|  |  | positive | **GynTect** | negative | 11 | 5 | 5 |  | 21 |
|  |  |  |  | positive | 4 | 0 | 2 |  | 6 |
|  |  |  | Total | | 15 | 5 | 7 |  | 27 |
|  |  | Total | **GynTect** | negative | 31 | 7 | 6 |  | 44 |
|  |  |  |  | positive | 5 | 0 | 2 |  | 7 |
|  |  |  | Total | | 36 | 7 | 8 |  | 51 |
|  | positive | negative | **GynTect** | negative | 3 | 2 |  |  | 5 |
|  |  |  | Total | | 3 | 2 |  |  | 5 |
|  |  | positive | **GynTect** | negative | 7 | 11 | 9 |  | 27 |
|  |  |  |  | positive | 0 | 3 | 14 |  | 17 |
|  |  |  | Total | | 7 | 14 | 23 |  | 44 |
|  |  | Total | **GynTect** | negative | 10 | 13 | 9 |  | 32 |
|  |  |  |  | positive | 0 | 3 | 14 |  | 17 |
|  |  |  | Total | | 10 | 16 | 23 |  | 49 |
|  | Total | negative | **GynTect** | negative | 24 | 4 | 1 |  | 29 |
|  |  |  |  | positive | 1 | 0 | 0 |  | 1 |
|  |  |  | Total | | 25 | 4 | 1 |  | 30 |
|  |  | positive | **GynTect** | negative | 20 | 18 | 14 | 0 | 52 |
|  |  |  |  | positive | 4 | 3 | 17 | 1 | 25 |
|  |  |  | Total | | 24 | 21 | 31 | 1 | 77 |
|  |  | Total | **GynTect** | negative | 44 | 22 | 15 | 0 | 81 |
|  |  |  |  | positive | 5 | 3 | 17 | 1 | 26 |
|  |  |  | Total | | 49 | 25 | 32 | 1 | 107 |

|  |  |  |  |  | **Histology** | | | | |
| --- | --- | --- | --- | --- | --- | --- | --- | --- | --- |
| **Age** | **Pap III+** | **HPV** |  |  | **noCIN** | **CIN 1-2** | **CIN 3** | **CxCa** | **Total** |
| >=30 y | missing | negative | **GynTect** | negative | 3 |  |  |  | 3 |
|  |  |  |  | positive | 2 |  |  |  | 2 |
|  |  |  | Total | | 5 |  |  |  | 5 |
|  |  | positive | **GynTect** | negative | 2 |  | 3 | 0 | 5 |
|  |  |  |  | positive | 1 |  | 2 | 2 | 5 |
|  |  |  | Total | | 3 |  | 5 | 2 | 10 |
|  |  | Total | **GynTect** | negative | 5 |  | 3 | 0 | 8 |
|  |  |  |  | positive | 3 |  | 2 | 2 | 7 |
|  |  |  | Total | | 8 |  | 5 | 2 | 15 |
|  | negative | negative | **GynTect** | negative | 56 | 2 | 0 |  | 58 |
|  |  |  |  | positive | 5 | 1 | 1 |  | 7 |
|  |  |  | Total | | 61 | 3 | 1 |  | 65 |
|  |  | positive | **GynTect** | negative | 17 | 6 | 2 |  | 25 |
|  |  |  |  | positive | 6 | 2 | 4 |  | 12 |
|  |  |  | Total | | 23 | 8 | 6 |  | 37 |
|  |  | Total | **GynTect** | negative | 73 | 8 | 2 |  | 83 |
|  |  |  |  | positive | 11 | 3 | 5 |  | 19 |
|  |  |  | Total | | 84 | 11 | 7 |  | 102 |
|  | positive | negative | **GynTect** | negative | 9 | 2 | 0 |  | 11 |
|  |  |  |  | positive | 4 | 0 | 2 |  | 6 |
|  |  |  | Total | | 13 | 2 | 2 |  | 17 |
|  |  | positive | **GynTect** | negative | 6 | 7 | 10 | 0 | 23 |
|  |  |  |  | positive | 2 | 6 | 32 | 2 | 42 |
|  |  |  | Total | | 8 | 13 | 42 | 2 | 65 |
|  |  | Total | **GynTect** | negative | 15 | 9 | 10 | 0 | 34 |
|  |  |  |  | positive | 6 | 6 | 34 | 2 | 48 |
|  |  |  | Total | | 21 | 15 | 44 | 2 | 82 |
|  | Total | negative | **GynTect** | negative | 68 | 4 | 0 |  | 72 |
|  |  |  |  | positive | 11 | 1 | 3 |  | 15 |
|  |  |  | Total | | 79 | 5 | 3 |  | 87 |
|  |  | positive | **GynTect** | negative | 25 | 13 | 15 | 0 | 53 |
|  |  |  |  | positive | 9 | 8 | 38 | 4 | 59 |
|  |  |  | Total | | 34 | 21 | 53 | 4 | 112 |
|  |  | Total | **GynTect** | negative | 93 | 17 | 15 | 0 | 125 |
|  |  |  |  | positive | 20 | 9 | 41 | 4 | 74 |
|  |  |  | Total | | 113 | 26 | 56 | 4 | 199 |

|  |  |  |  |  | **Histology** | | | | |
| --- | --- | --- | --- | --- | --- | --- | --- | --- | --- |
| **Age** | **Pap III+** | **HPV** |  |  | **noCIN** | **CIN 1-2** | **CIN 3** | **CxCa** | **Total** |
| Total | missing | negative | **GynTect** | negative | 4 |  |  |  | 4 |
|  |  |  |  | positive | 2 |  |  |  | 2 |
|  |  |  | Total | | 6 |  |  |  | 6 |
|  |  | positive | **GynTect** | negative | 4 | 2 | 3 | 0 | 9 |
|  |  |  |  | positive | 1 | 0 | 3 | 3 | 7 |
|  |  |  | Total | | 5 | 2 | 6 | 3 | 16 |
|  |  | Total | **GynTect** | negative | 8 | 2 | 3 | 0 | 13 |
|  |  |  |  | positive | 3 | 0 | 3 | 3 | 9 |
|  |  |  | Total | | 11 | 2 | 6 | 3 | 22 |
|  | negative | negative | **GynTect** | negative | 76 | 4 | 1 |  | 81 |
|  |  |  |  | positive | 6 | 1 | 1 |  | 8 |
|  |  |  | Total | | 82 | 5 | 2 |  | 89 |
|  |  | positive | **GynTect** | negative | 28 | 11 | 7 |  | 46 |
|  |  |  |  | positive | 10 | 2 | 6 |  | 18 |
|  |  |  | Total | | 38 | 13 | 13 |  | 64 |
|  |  | Total | **GynTect** | negative | 104 | 15 | 8 |  | 127 |
|  |  |  |  | positive | 16 | 3 | 7 |  | 26 |
|  |  |  | Total | | 120 | 18 | 15 |  | 153 |
|  | positive | negative | **GynTect** | negative | 12 | 4 | 0 |  | 16 |
|  |  |  |  | positive | 4 | 0 | 2 |  | 6 |
|  |  |  | Total | | 16 | 4 | 2 |  | 22 |
|  |  | positive | **GynTect** | negative | 13 | 18 | 19 | 0 | 50 |
|  |  |  |  | positive | 2 | 9 | 46 | 2 | 59 |
|  |  |  | Total | | 15 | 27 | 65 | 2 | 109 |
|  |  | Total | **GynTect** | negative | 25 | 22 | 19 | 0 | 66 |
|  |  |  |  | positive | 6 | 9 | 48 | 2 | 65 |
|  |  |  | Total | | 31 | 31 | 67 | 2 | 131 |
|  | Total | negative | **GynTect** | negative | 92 | 8 | 1 |  | 101 |
|  |  |  |  | positive | 12 | 1 | 3 |  | 16 |
|  |  |  | Total | | 104 | 9 | 4 |  | 117 |
|  |  | positive | **GynTect** | negative | 45 | 31 | 29 | 0 | 105 |
|  |  |  |  | positive | 13 | 11 | 55 | 5 | 84 |
|  |  |  | Total | | 58 | 42 | 84 | 5 | 189 |
|  |  | Total | **GynTect** | negative | 137 | 39 | 30 | 0 | 206 |
|  |  |  |  | positive | 25 | 12 | 58 | 5 | 100 |
|  |  |  | Total | | 162 | 51 | 88 | 5 | 306 |
